# Reconstructing the spread of measles in the 20th century: an epidemiological analysis of the period prior to the introduction of vaccination in Switzerland

**DOI:** 10.1093/aje/kwaf167

**Published:** 2025-08-11

**Authors:** Cyrill Friedauer, Katarina L Matthes, Phung Lang, Kaspar Staub

**Affiliations:** Institute of Evolutionary Medicine, University of Zurich, Zurich, Switzerland; Institute of Evolutionary Medicine, University of Zurich, Zurich, Switzerland; Epidemiology, Biostatistics and Prevention Institute, University of Zurich, Zurich, Switzerland; Institute of Evolutionary Medicine, University of Zurich, Zurich, Switzerland

**Keywords:** historical epidemiology, morbiditymortality, time series, long-term trend

## Abstract

In the early 20th century, measles posed a much greater threat to children than it does today in the present age of effective vaccination. The aim of this study is to quantify historical measles-related mortality, morbidity, and case fatality rate in Switzerland to better understand the dynamics of the spread prior to the introduction of vaccination. Historical measles case and death records were transcribed and digitized for the first time, drawing on official federal periodicals. The data were visualized and subsequently subjected to time series and wavelet analyses. Between 1876 and 2022, a total of 19 226 measles deaths were recorded in Switzerland. Children under five had the highest mortality rates. The 5-year average mortality rate peaked at 20.6 deaths per 100 000 inhabitants at the beginning of the 20th century, subsequently falling to 0.7 by the 1940s. After the introduction of vaccination in the 1970s, mortality rates remained steadily below 0.01. A notable decrease in measles incidence was not observed until the 1960s. Time series analysis revealed annual cycles indicating seasonality, which were embedded in longer cycles, extending from 1.5 to 5 years. These findings provide insight into the pre-vaccine era and highlight the importance of high immunization and vaccination rates.

## Introduction

At the beginning of the 20th century, infectious diseases were a pervasive health threat and a major cause of death worldwide. In the United States, almost a quarter of all deaths in 1900 were due to infectious diseases.^1^ At that time, children and infants were particularly vulnerable, with infectious diseases accounting for 61.6% of all child deaths.^1^ However, the epidemiological transition framework indicates that this situation has changed markedly over the last century, especially in high-income countries: infectious diseases have been replaced by noncommunicable diseases as the primary cause of morbidity and mortality.^2-5^ The mortality rate due to infectious diseases in the United States fell from 797 per 100 000 to 36 between 1900 and 1990.^3^ Many factors contributed to this decrease, including improved nutritional status and housing, hygiene standards and advances in medical treatment.^3^^,^^6^ Additionally, mass immunization programs throughout the 20th century subsequently played a key role in the eradication and control of infectious diseases.^6^^,^^7^ It is estimated that modern immunization programs for measles, polio and DTP (diphtheria–tetanus–pertussis) prevent about 2.5 million child deaths (<5 years) worldwide each year.^8^

One particularly common and deadly disease at the beginning of the 20th century was measles.^9^ Measles virus infection typically presents with high fever, maculopapular rash with cough, rhinitis and conjunctivitis after an incubation period of 10 to 14 days.^10^^,^^11^ While some measles cases make a full recovery, others develop life-threatening complications and secondary infections.^9^^,^^12^ Thus, measles remains a potentially lethal infection today, particularly in the immunocompromised, and the WHO’s aim of eliminating it by 2030 is far from complete.^13^ At present, estimates for the case fatality ratio vary widely from under 0.01% to over 5%. This variation is, among a number of factors, attributable to vaccine coverage and the regional healthcare situation.^10^ Recent outbreaks in the U.S. for example, show a case fatality ratio of 0.32% with 3 deaths and a total of 935 cases until the May 1, 2025. Notably, 96% of the cases occurred in patients which were unvaccinated or with unknown vaccination status.^14^ The most common complications are pneumonia and diarrhea, both of which can lead to death.^15^ The measles virus is transmitted through the respiratory tract by droplets or aerosols that can remain in the air for several hours.^16^^,^^17^ It is one of the most infectious pathogens, with a high transmissibility and a basic reproduction number (R0) of approximately 12 to 18.^11^^,^^18^ This means that it has the potential to spread rapidly when immunization levels are low.^11^ In a densely populated environment where vaccination coverage is low, the highest incidence is found in children and infants, because they have not yet been immunized, be it through infection, as was usually the case in the past, or, more recently, through vaccination.^10^ Since successful recovery from measles confers lifelong immunity comparable to vaccination, measles is predominantly a childhood disease.^11^^,^^19^

A study from the United States shows that from 1888 to 2011, measles typically occurred in annual seasonal epidemics.^20^ However, these annual epidemics were embedded in longer-term cycles of 2 to 5 years, as shown by data for England and Wales.^21^^,^^22^ These cyclical peaks are the result of the fluctuating number of susceptible hosts in the population. Thus, high birth rates lead to an accumulation of susceptible individuals. Conversely, high levels of immunization following an outbreak or as the result of vaccination reduce the number of susceptible individuals.^21^^,^^22^ In addition, there are several other elements that play a role in the cyclical course of the disease, including seasonality, with measles outbreaks in the northern hemisphere typically occurring in the late winter and early spring months, and social factors, such as the timing of school holidays (potentially reducing contacts), which affect the number of transmission opportunities.^21^^,^^22^ Because the measles virus has no animal reservoir and cannot persist as a latent infection, there must be an unbroken chain of infection for the virus to remain in circulation.^21^ This means that measles may vanish locally in a population with a very high immunization rate, while, conversely, in a population with many susceptible hosts, there exists the potential for a new outbreak.^22^ Spatial heterogeneity in susceptibility leads to the emergence of so-called traveling waves of measles (ie, the spatial–temporal propagation of the disease across a population, from a localized origin to the surroundings), which have been well documented historically, for example in the United Kingdom.^22^

In 1963, a vaccine against measles was licensed for the very first time in the United States. Before it became available, almost all children contracted measles, and there were an estimated 135 million cases per year worldwide, of which more than 6 million were measles-related deaths.^9^ In Switzerland, the first vaccination was officially recommended by the Federal Office of Public Health in 1976 for children aged 12 months.^23^ This was followed by the first combined MMR (measles, mumps, rubella) vaccine in 1985.^23^ The result was stable vaccination coverage of about 80% in children under 2 years of age between 1987 and 2002.^23-25^ Nevertheless, probably due to insufficient vaccination coverage, various measles outbreaks were recorded in Switzerland between 2003 and 2011, which, in the following years, led to increased vaccination and public health communication efforts at various levels.^26-28^ In 2022, coverage has increased to 94% in 2-year-olds and 96% in 16-year-olds (for two doses) as recent data from the Swiss Vaccination Coverage Survey shows.^23-25^^,^^29^^,^^30^

Studies from the United States, Netherlands, and Italy have compared long-term and annual historical trends in the incidence of certain childhood diseases, including measles, mainly in the context of the introduction of vaccination programs during the 20th century.^3^^,^^7^^,^^20^^,^^31^ Although these studies give us the big picture and compare different infectious diseases, including measles, they do not go into great depth on the temporal dimension of individual diseases. One of the few studies that specifically models and compares measles outbreaks in the pre-vaccination and vaccination periods focuses on England and Wales between 1944 and 1994,^22^ but, to our knowledge, there are no such detailed studies on measles outbreaks for other European countries.

Moreover, the course of measles in Switzerland and the Swiss cantons during the 20th century has not been investigated in depth, because detailed archival data was not previously accessible.^6^ We are now making this archival data accessible for the first time in digital form. To date, there has been no quantification of the disease burden and transmission dynamics of measles in Switzerland in the course of the 20th century, prior to the introduction of mass vaccination in the 1970s. Filling this research gap will help us to understand the history of measles in Switzerland and provide valuable information about disease dynamics. In addition, quantifying the burden of the disease makes its scale in the 20th century more concrete, thus providing an important foundation for discussions in the face of the current trends in vaccine hesitancy.

The aim of this study is to quantify the mortality and morbidity burden of measles in Switzerland throughout the 20th century and to gain a better understanding of the regional and temporal dynamics of spread prior to the introduction of the vaccine in 1976.^23^ Specifically, we will reconstruct the annual mortality burden by age and case–fatality ratio, as well as the monthly incidence, in order to describe seasonality and other cyclical dynamics in selected cantons.

## Methods

### Data sources

This study is based on historical archival data published by the Swiss Federal Office of Public Health (FOPH) and the Federal Statistical Office (FSO). The following data have been digitized for the first time from historical publications: (1) mortality data: yearly reported measles deaths for the period 1875 to 2022 were obtained from the weekly bulletin published by the FOPH, as well as the causes of death statistics from the FSO (*Statistik der Todesursachen*). In addition, yearly data on measles mortality by age group were also obtained from the cause of death statistics from the FSO; (2) morbidity data: monthly reported measles cases for all cantons for the period 1910 to 1973 were obtained from the weekly bulletin published by the FOPH. For the period 1988 to 2022, these data were provided directly by the FOPH, but no morbidity data are available for Switzerland for the period 1974 to 1987.

For reasons related to mandatory reporting requirements prior to 1974, we have restricted the morbidity analysis to seven selected cantons with documented mandatory reporting requirements (more details below). Historical population figures for the permanent residents of each canton were obtained from the Historical Statistics of Switzerland Online (HSSO). Annual life tables, age composition and total mortality figures in Switzerland were obtained from the Human Mortality Database.

### Reporting and data quality

Annual mortality statistics, including age, were provided by the Federal Statistical Office for the period 1875 to 2022. Mortality data are not affected by differences in cantonal legislation, as measles has been part of Switzerland’s official nationwide causes of death catalogue since 1876.^32^^,^^33^ However, the quality of the reporting at the beginning of our time series is assumed to be of poor quality for several reasons.^32^ First, reporting by doctors was not standardized and their handwriting was often illegible. In addition, the legal requirement for doctors to report causes of death was not clearly stated, remaining a vaguely worded rule. As a result, there was insufficient compliance among doctors in many cantons.^32^ This situation improved considerably in the following years, thanks to the ambitions of the Federal Statistical Office. In 1901, for example, the nomenclature of causes of death was completely revised and standardized.^34^^,^^32^ As a result of the continuous improvements made by the Federal Statistical Office, the quality of the mortality data for the 20th century steadily improved.^35^

In Switzerland, the spread of measles was monitored by authorities in various ways throughout the 20th century. Nationwide mandatory reporting of new cases was introduced for the first time in 1943.^36^ Previously, several cantons had introduced mandatory reporting, including Zurich (1879), Bern (1898), Lucerne (1877), St Gallen (1902), Basel City (1875), Grisons (1889) and Geneva (1891).^37-43^ Given their long-standing existence of a legal obligation to report measles cases, our research focused primarily on these seven cantons. This selection was intended to provide a representative sample of the different regions of Switzerland and to reduce the risk of underreporting. In 1974, there was a change in case-reporting policy and the measles cases were grouped under the term “exanthematous diseases”.^44^ As a result, measles disappeared as an individual disease from the weekly reporting until 1988, after which measles-specific data became available again in the weekly bulletin of the FOPH. As a result, there is unfortunately a data gap for this period. From 1989 onwards, measles was listed separately again, but it was only after 1999 that a reporting obligation specifically for measles was reintroduced.^45^ Despite this reporting requirement, some degree of underreporting must be assumed.^28^^,^^46^ Consequently, these figures can never show the full extent of the disease burden. However, they are useful for analyzing spatial and temporal transmission dynamics and periodicity of outbreaks.

### Missing values

There were seven isolated months between 1913 and 1948 for which reported-case values were completely missing for all the cantons. These were imputed by simple linear interpolation. In contrast, there were no missing values for reported deaths. Furthermore, a notable portion of the monthly reported case numbers was additionally annotated with the textual remark “epidemic” (*n* = 805 out of 19 288, or 4.2%), indicating the possibility of additional unreported cases. In these cases, we assumed a minimal scenario involving only the reported numerical case numbers and disregarding the additional textual information “epidemic”.

### Statistical analysis

Mortality and morbidity are always presented in relation to the population of Switzerland or the selected cantons, as well as for age groups, if applicable.

In order to decompose and visualize the monthly incidence data for Switzerland and the selected cantons, we first computed a time-series analysis for the seven selected cantons. As conventional spectral analysis requires stationary data, we performed a wavelet time-series analysis for each canton using the “WaveletComp” package for R.^47^^,^^48^ A wavelet time-series analysis can describe periodic phenomena of a nonstationary time series.^49^^,^^50^ Because we were interested in the periodical behavior of measles incidence in general, over time and in relation to particular cantons, we also implemented the wavelet analysis for each of the selected cantons, as well as a cross-wavelet analysis to check the synchronicity between each pair of cantons. For this purpose, the Morlet wavelet shape was used. Statistical significance was evaluated using a simulation algorithm that generates surrogate times series consisting of white noise.^50^ The significance level was chosen at alpha = 0.05. For the average wavelet power, we have extracted the local maxima. To assess how the signal strength behaved over time, we have presented wavelet power spectra and interpreted the results visually.

R (version 4.2.0) was used for statistical analysis.^48^

## Results

A total of 19 226 measles deaths were reported in Switzerland between 1876 and 2022. The annual mortality averages calculated over 5 years are shown in Table 1. In the period 1877 to 1899, the mortality rate fluctuated between 9.4 (95% CI, 8.9-9.9) and 13.1 (95% CI, 12.5-13.7) per 100 000 inhabitants, followed by a peak at the beginning of the 20th century, from 1900 to 1904, with a mortality rate of 20.6 (95% CI, 19.9-21.3) per 100 000. Subsequently, there was a rapid decline of the mortality burden at the beginning of the century, falling to an average mortality rate of 0.7 (95% CI, 0.6-0.8) per 100 000 in the period 1940 to 1944. This represents a reduction to 3.3% (CI, 2.7%-4%) of the level in 1900 to 1904. Towards the end of the 20th century, and in particular after 1980 (the measles vaccine was first recommended in 1976), the mortality rate remained below 0.01 per 100 000 population.

**Table 1:** Average annual mortality for measles over 5-year periods for Switzerland (per 100 000 inhabitants), as well as annual averages of mortality (per 100 000 inhabitants), incidence (per 100 000 inhabitants) and case-fatality ratio (%) over 5 years for the selected cantons of Zurich, Bern, Lucerne, St Gallen, Grisons, Basel-City and Geneva.

**Year**	**Mortality Switzerland**	**Mortality selected cantons (95% CI)**	**Incidence selected cantons (95% CI)**	**Case fatality ratio selected cantons (95% CI)**
1877-1879	11.15 (10.45-11.89)	11.77 (10.76-12.85)		
1880-1884	9.85 (9.34-10.38)	10.05 (9.34-10.80)		
1885-1889	13.11 (12.53-13.72)	13.85 (13.02-14.72)		
1890-1894	18.2 (17.52-18.9)	19.16 (18.21-20.16)		
1895-1899	9.40 (8.93-9.89)	9.86 (9.21-10.56)		
1900-1904	20.59 (19.91-21.29)	19.58 (18.67-20.52)		
1905-1909	13.83 (13.29-14.39)	12.41 (11.72-13.14)		
1910-1914	8.28 (7.87-8.69)	6.22 (5.75-6.72)	219.60 (216.75-222.47)	2.83 (2.62-3.06)
1915-1919	4.23 (3.95-4.53)	4.68 (4.28-5.11)	237.38 (234.45-240.34)	1.97 (1.80-2.15)
1920-1924	4.27 (3.98-4.57)	3.14 (2.81-3.50)	232.35 (229.45-235.27)	1.35 (1.21-1.50)
1925-1929	2.92 (2.69-3.17)	2.56 (2.27-2.88)	223.22 (220.41-226.05)	1.15 (1.02-1.29)
1930-1934	1.55 (1.38-1.73)	1.38 (1.17-1.61)	223.34 (220.60-226.11)	0.62 (0.52-0.72)
1935-1939	0.83 (0.71-0.96)	0.74 (0.60-0.92)	125.88 (123.84-127.94)	0.59 (0.47-0.73)
1940-1944	0.68 (0.58-0.80)	0.55 (0.42-0.70)	217.57 (214.93-220.24)	0.25 (0.19-0.32)
1945-1949	0.36 (0.28-0.44)	0.36 (0.26-0.48)	201.40 (198.93-203.90)	0.18 (0.13-0.24)
1950-1954	0.37 (0.30-0.46)	0.27 (0.19-0.38)	250.18 (247.52-252.86)	0.11 (0.08-0.15)
1955-1959	0.20 (0.14-0.26)	0.15 (0.10-0.23)	181.46 (179.28-183.67)	0.08 (0.0-0.13)
1960-1964	0.14 (0.10-0.19)	0.13 (0.08-0.19)	193.54 (191.38-95.71)	0.07 (0.04-0.10)
1965-1969	0.09 (0.06-0.13)	0.06 (0.03-0.11)	141.62 (139.83-143.42)	0.04 (0.02-0.08)
1970-1974	0.06 (0.04-0.10)	0.05 (0.02-0.10)	86.15 (84.79-87.53)	0.06 (0.03-0.11)
1975-1979	0.03 (0.01-0.05)	0.01 (0.00-0.03)		
1980-1984	0.01 (0.00-0.03)	0.02 (0.00-0.05)		
1985-1989	0.01 (0.00-0.02)	0.01 (0.00-0.03)		
1990-1994	0.00 (0.00-0.02)	0.00 (0.00-0.02)	1.29 (1.13-1.46)	0.00 (0.00-1.55)
1995-1999	0.00 (0.00-0.02)	0.01 (0.00-0.03)	1.46 (1.30-1.65)	0.36 (0.01-2.03)
2000-2004	0.00 (0.00-0.01)	0.00 (0.00-0.02)	1.44 (1.28-1.62)	0.00 (0.00-1.33)
2005-2009	0.00 (0.00-0.01)	0.00 (0.00-0.02)	12.39 (11.91-12.89)	0.00 (0.00-0.15)
2010-2014	0.00 (0.00-0.01)	0.00 (0.00-0.02)	2.32 (2.12-2.53)	0.00 (0.00-0.76)
2015-2019	0.00 (0.00-0.02)	0.01 (0.00-0.03)	1.09 (0.96-1.24)	0.83 (0.10-3.01)
2020-2023	0.00 (0.00-0.01)	0.00 (0.00-0.03)	0.35 (0.26-0.47)	

**Figure 1:**
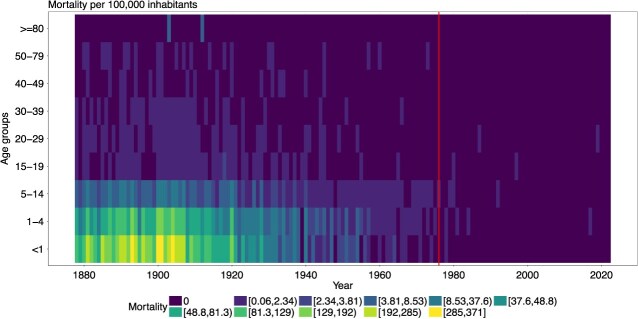
Yearly measles mortality per 100 000 population by age group for Switzerland. The vertical reference line indicates 1976, when the first measles vaccination was recommended.

The distribution of measles-specific deaths by age group is shown in Figure 1. Children under 5 years of age are clearly the most affected age group. Consistent with the overall mortality rate, a peak can be observed at the very beginning of the century, particularly in the age groups < 1 year and 1 to 4 years. In 1900, the mortality rate was 338.6 per 100 000 (95% CI, 300.2-380.5) in the age group < 1 year and 147.7 per 100 000 (95% CI, 134.2-162.2) in the age group 1 to 4 years. By 1940, mortality in these two age groups had fallen in line with overall mortality. For < 1-year-olds, the mortality rate was 14.6 (95% CI, 6.7-27.6) and for 1- to 4-year-olds 20.5 (95% CI, 15.2-27.0). After 1980, there were very few isolated death cases in these two age groups.

**Figure 2:**
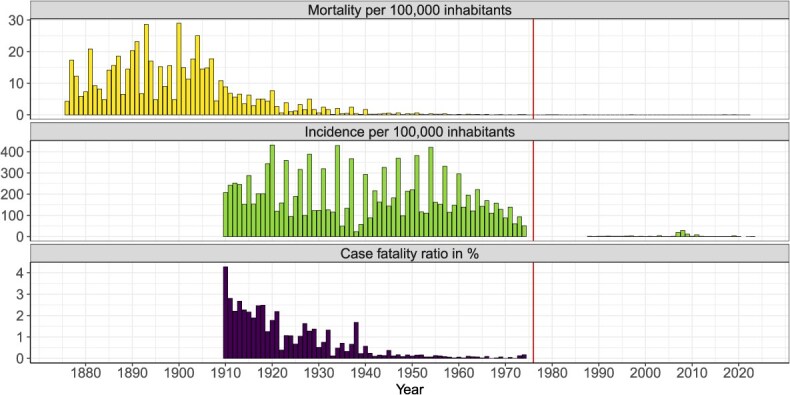
Yearly measles mortality (per 100 000 inhabitants), incidence (per 100 000 inhabitants) and case-fatality ratio (%) of all seven selected cantons combined (Zurich, Bern, Lucerne, St Gallen, Grisons, Basel-City and Geneva). The vertical reference line indicates 1976, when the first measles vaccination was recommended. Due to the small numbers of cases and deaths we have not shown the case-fatality ratio in the figure after 1974.

In terms of morbidity, in the period 1910 to 1974, a total of 317 667 cases were reported in the seven selected cantons with documented mandatory reporting (Zurich, Bern, Lucerne, St Gallen, Grisons, Basel City, and Geneva) and 470 476 for Switzerland as a whole. Unlike mortality, the incidence in the selected cantons did not decline immediately at the beginning of the 20th century, as Table 1 and Figure 2 show. Except for the period 1935 to 1939, the 5-year average incidence remained above the rate of 200 new cases per 100 000 inhabitants from 1910 to 1954. The years with slightly lower incidences of measles around 1940 correlate visually with a temporary low in the birth rate in Switzerland around the same time (Figure S1). From 1950 to 1954, the 5-year average incidence increased again, even reaching a maximum of 250.2 (95% CI, 247.5-252.9). By this time, the mortality rate had already declined from 6.2 (95% CI, 5.8-6.7) to 0.3 (95% CI, 0.2-0.4), a reduction of more than 95%. For this reason, the average case-fatality rate also decreased from 2.8 (95% CI, 2.6-3.1) in 1910 to 1914 to 0.1 (95% CI, 0.1-0.2) in 1950 to 1954. During the second half of the 1960s, the incidences began to decline again, which in turn was visually correlated with a decline in the birth rate (Figure S1). After 1974, no incidence data are available for measles until 1988. During this period, a further rapid decline can be assumed, as the average incidence in 1990 to 1994 was only 1.3 (95% CI, 1.1-1.5).

**Figure 3:**
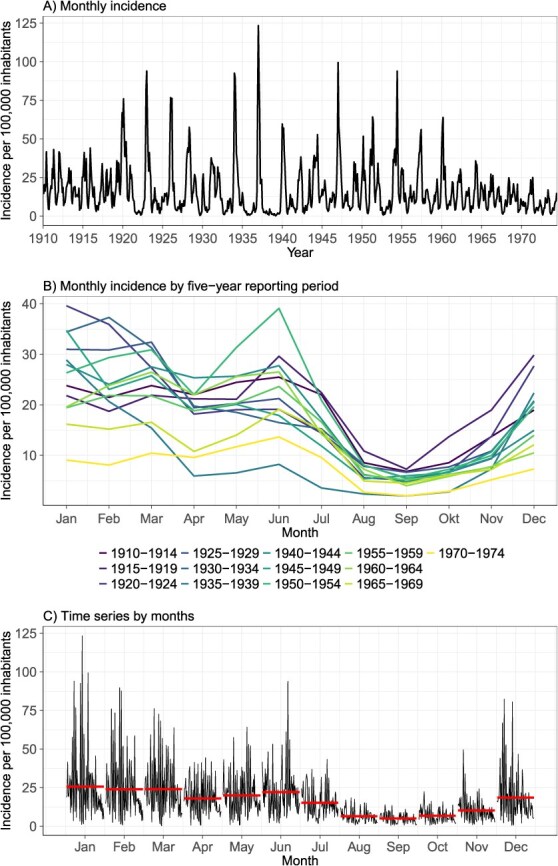
(A) Time series of monthly measles incidence, (B) monthly measles incidence by 5-year reporting period and (C) measles time series by individual months. The horizontal reference lines indicate the average over the entire time period (1910-1974). All figures are based on the data of the selected cantons combined (Zurich, Bern, Lucerne, St Gallen, Grisons, Basel-City and Geneva).

Figure 3A provides further insight based on monthly morbidity data for the selected cantons combined (the monthly numbers for each of the selected cantons are shown individually in Figure S2), which clearly demonstrates that the incidence reached large peaks following a more or less regular pattern (see below). Figure 3B illustrates the monthly seasonality of measles for several different time periods. Figure 3C depicts the time series for each month, with the red line representing the average incidence over the total observed time range. The lowest measles incidence is usually observed in the late summer months of August and September. From October onwards, the incidence increases, peaking between January and March.

**Figure 4:**
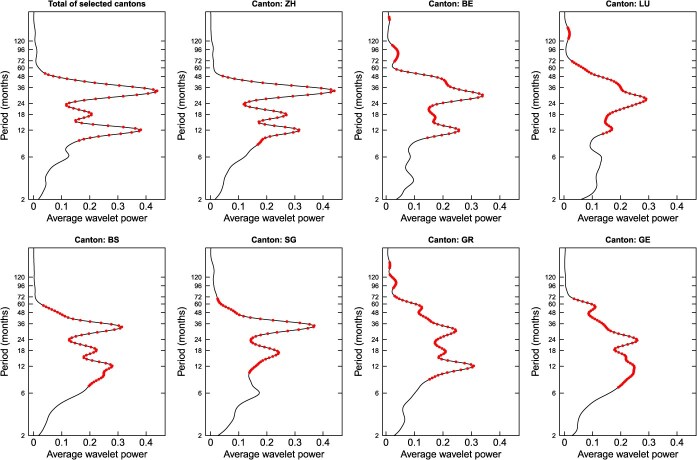
Average power of the wavelet analysis (over the entire observation period) of measles incidence in the selected cantons of Zurich (ZH), Bern (BE), Lucerne (LU), St Gallen (SG), Grisons (GR), Basel-City (BS) and Geneva (GE) individually, as well as the total for the selected cantons. The dots indicate significance, at a significance level of alpha = 0.05 (*P* < 0.05). The y-axis follows a logarithmic progression.

Furthermore, we examined the periodic behavior of the time series of measles incidence across individual Swiss cantons. Figure 4 shows the average wavelet power analysis (ie, the average wavelet power over the entire observation period) for each individual canton and jointly for all the selected cantons. If we look at the time series with the total of the selected cantons, the periods with the strongest signals (local maxima) were observed at 12.1, 18.4 and 33.1 months. The strong signal at 12.1 months suggests that this time series followed an annual cycle. However, the even stronger signal with the maximum at 33.1 months implies a superimposed cycle of approximately 3 years. At the level of each individual canton, all the cantons except St. Gallen show this clear peak in signal strength around approximately 12 months (Figure 4), underlining that measles outbreaks followed this strong annual cycle in most of the cantons. Furthermore, all cantons show more than just one peak in signal strength and there were several more significant wavelet power peaks between 18 and 60 months for the individual cantons. This suggests that most cantons show, besides the annual cycle, also longer cyclical behavior between approximately 1.5 and 5 years.

The cantons of Zurich and Basel City exhibit very similar periodic patterns, with dominant peaks at 12.1, 17.8 and 33.1 months for Zurich and at 12.1, 18.4, 33.1 for Basel City. In contrast, Geneva has a more unique pattern, with peaks at 11.7, 14.9, 23.4 and 55.7 months. These differences are also apparent in the cross-wavelet power calculations between cantons (Figure S4, Figure S5). Geneva, which is located in the southwest of Switzerland and is geographically most distant from the other cantons, shows the lowest average cross-wavelet power.

**Figure 5:**
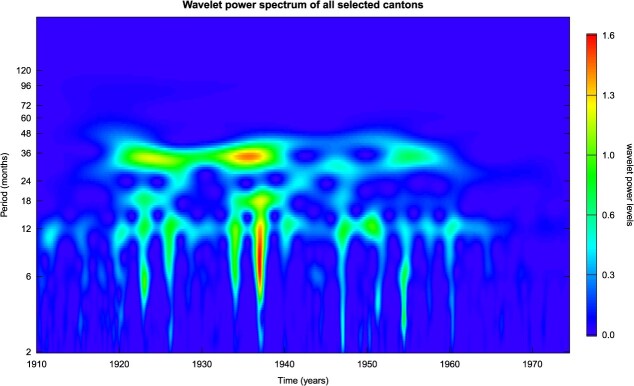
Wavelet power spectrum of monthly measles incidence for the total of the selected cantons. The y-axis follows a logarithmic progression. The color scale indicates the signal strength.

Figure 5 shows the wavelet power spectrum of the time series for the total of the selected cantons. The power spectrum includes the temporal information of the periodic behavior in contrast to the average wavelet power in Figure 4. The colors indicate that the signal strength around the 12-month period remains relatively constant over the entire observation period. The signal strength around the 18-month period is only intermittent with occasional strong signals, especially in the 1920s and late 1930s. The signal at around the 33-month period is relatively constant from 1920 to 1960 but temporarily reduced between 1940 and 1950 (Figure S3 shows the individual wavelet power spectra for the individual cantons).

## Discussion

Our study has shown that there was a remarkable decline in the disease burden of measles in Switzerland throughout the 20th century and in particular after the introduction of vaccination in the 1970s. Mortality and case-fatality rates fell in the first half of the 20th century, whereas the decline in average morbidity lagged behind and was not observed until the 1960s. It was only with the introduction of the vaccination program in the 1970s that both mortality and morbidity were successfully reduced to a very low level and stabilized. In the pre-vaccine era from 1910 to 1974, the morbidity of measles showed strong seasonality, as well as cyclical behavior over longer periods. The length of these cycles varied across the cantons examined, with some of them showing very similar patterns, while others display more unique ones.

The decline of measles-specific mortality among all age groups in Switzerland began early in the 20th century. A similar reduction in the early 20th century has also been demonstrated for other countries, like the Netherlands, the United States and Italy.^3^^,^^7^^,^^31^ According to the literature, the most likely explanation for this decline is higher standards of living, better nutrition and better overall healthcare conditions.^3^^,^^31^ In addition, the improved treatment of secondary infections may have played an important role in the decline of mortality rates.^31^ Measles often became fatal due to secondary infections like pneumonia,^12^ partly because of its immunosuppressive effects.^11^ However, from 1900 on we did not see a decline in 5-year average incidence until the 1960s. Consistent with this, the case–fatality ratio declined from 1910 to roughly 1940.

Our data show clear seasonality in measles cases, with a peak in the winter months of January and February, which aligns with the consensus in the literature.^21^ The lowest incidence is seen in the late summer months of August and September. The same patterns were observed in the pre-vaccine era in the United States and England.^51^^,^^52^ Possible explanations for this include the presence of factors that facilitate the transmission of measles. For example, several hypotheses have been advanced in the literature about direct pathways through which temperature could affect the transmission of respiratory viruses.^53^^,^^54^ Whether this is also true for measles remains an open question. Furthermore, it has been hypothesized that the patterns of school holidays influence the spread of measles via the accumulation of many susceptible individuals at the start of the school year.^21^^,^^51^^,^^52^

In accordance with the findings in the literature, these seasonal changes in Switzerland were embedded in longer cycles with a period of 1.5 to 5 years. Studies from Italy and the United States similarly describe periods of 2 to 5 years.^3^^,^^7^ In theory—and according to the literature—these cycles may result from the fluctuating number of susceptible hosts available, which is influenced by various factors, including the birth rate, internal/external migration (eg, urbanization) and the immunization level in a population.^51^ For Switzerland, we can observe a visual correlation between the incidence of measles and the birth rate, which should be investigated in follow-up studies.^55^ The periods of these cycles varied clearly among the selected cantons: on the one hand, we found certain similarities among geographically close cantons and cantons with densely populated urban centers, such as Zurich, Bern, and Basel City. On the other hand, we observed distinct differences among the cantons, when the signal strength of the periods was different in distant cantons, such as Geneva. Similarly, the major population centers in England and Wales exhibited a synchronized biennial pattern from 1950 to 1967, suggesting that these centers behaved as an epidemiological unit, while less densely populated regions tended not to display the same pattern as the population centres.^51^ However, the regional and temporal patterns could not be fully explained. This finding aligns with the literature, where traveling waves have been observed, but a degree of randomness remains.^22^ In our case, it was difficult to detect traveling waves due to the lack of accuracy in the temporal and especially in the spatial resolution of our data.

Furthermore, from the 1960s onwards, the cyclic peaks became less pronounced, and we must assume a further reduction of the measles incidence rate from 1970 to 1990, since we once again observe a very low incidence rate in the 1990s. Unfortunately, however, there is no case reporting data available for the period 1974 to 1988. As a result, we cannot assess the disease dynamics in Switzerland for this period and analyze when and how cyclic epidemic peaks disappeared. It is therefore impossible to precisely capture the effect of immunization programs in Switzerland, which started during this period. However, the vaccine did stabilize the incidence and mortality rates in Switzerland at a very low level, as was the case in Italy and the Netherlands.^7^^,^^31^ The recent measles outbreaks in the United States and Europe show that the goal of eliminating measles will not be achieved by 2030, as set by the WHO. This is particularly concerning because measles continues to be a potentially lethal infection, especially in immunocompromised persons.^13^^,^^56^

Our study has several limitations: first, we rely on aggregated rather than individual data, which in our case do not include potential risk factors at the individual level, such as sex, age, and socioeconomic background. Thus, on the theoretical level, there is a risk of committing an ecological fallacy (the error of attributing the characteristics of a population to an individual). Second, we must assume a certain level of underreporting with regard to measles-related deaths, since it is unclear whether deaths from secondary infections were always reported as measles deaths. We therefore suspect that the actual mortality rate may have been even higher. Third, although reporting was mandatory, we must assume the morbidity data to be underreported as well. In addition, certain case numbers were additionally marked with the text “epidemic outbreak”, suggesting additional cases, but no actual numbers were provided. In these rare instances, we had to assume a minimal scenario and use the numbers provided. All this suggests that we should assume that the actual incidence figures were higher than those observed in our analysis. Fourth, despite our best efforts to search the archives, we were unable to resolve the incidence data gap between 1974 and 1989. This means that the period during which the vaccine became increasingly widespread is not covered.

In conclusion, thanks to newly digitized data, we are able to show for the first time that there were regular outbreaks of measles in the pre-vaccination era prior to 1976 in Switzerland and that these outbreaks followed certain periodic patterns. Although the mortality rate for measles had already declined at the beginning of the 20th century, the morbidity burden remained high until around the middle of the 20th century. It was only with the introduction of the vaccination program in the 1970s that both mortality and morbidity were successfully reduced to a very low level and stabilized. Such historical insights from a time before the introduction and widespread use of effective vaccination demonstrate the health burden and outbreak dynamics connected to measles to which the Swiss population was exposed. This experiential knowledge has been increasingly lost in Switzerland, and the results of our study aim to raise awareness of this potentially recurring public health threat, especially given the rise in vaccine hesitancy, because measles is far from being eliminated and continues to be a potentially lethal infection.

## Ethics

As this study was based solely on historical epidemiological data and did not involve any human subjects, human material or personal data, no approval was required from the cantonal ethics committee.

## Supplementary Material

Web_Material_kwaf167

## Data Availability

The data and code underlying this manuscript are publicly available via GitHub: https://github.com/friedacy/Measles-Switzerland.git.
