# Supplementary material for: Reconstructing the spread of measles in the 20th century: an epidemiological analysis of the period prior to the introduction of vaccination in Switzerland
Source: Am J Epidemiol. 2025 Aug 11;194(12):3556–65. doi: 10.1093/aje/kwaf167 (PMC12671959; doi:10.1093/aje/kwaf167)

**SUPPLEMENTARY MATERIAL**

**Reconstructing the spread of measles in the 20th century: An epidemiological analysis of the period prior to the introduction of vaccination in Switzerland**

Cyrill Friedauer ^1^, Katarina L Matthes ^1^, Phung Lang ^2^, Kaspar Staub ^1^ *

^1^ Institute of Evolutionary Medicine, University of Zurich, Switzerland

^2^ Epidemiology, Biostatistics and Prevention Institute, University of Zurich, Switzerland

**List of included materials:** Figures S1-S5

Figure S1: The annual measles incidence (A) and mortality figures (B) for Switzerland from 1910 to 1970 compared to the proportion of the population under 5 years of age (C), the crude birth rate (D), the absolute number of births (E) and GDP per capita (F). Source: Human Mortality Database and Floris et al. 2019

**
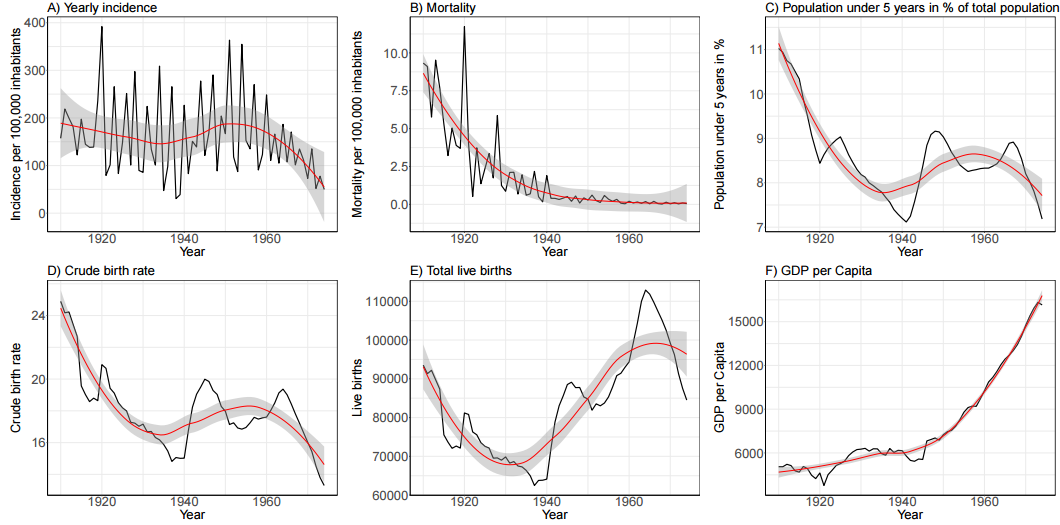
**

Figure S2: Monthly measles incidence in the selected cantons by decade between 1910 and 1970.


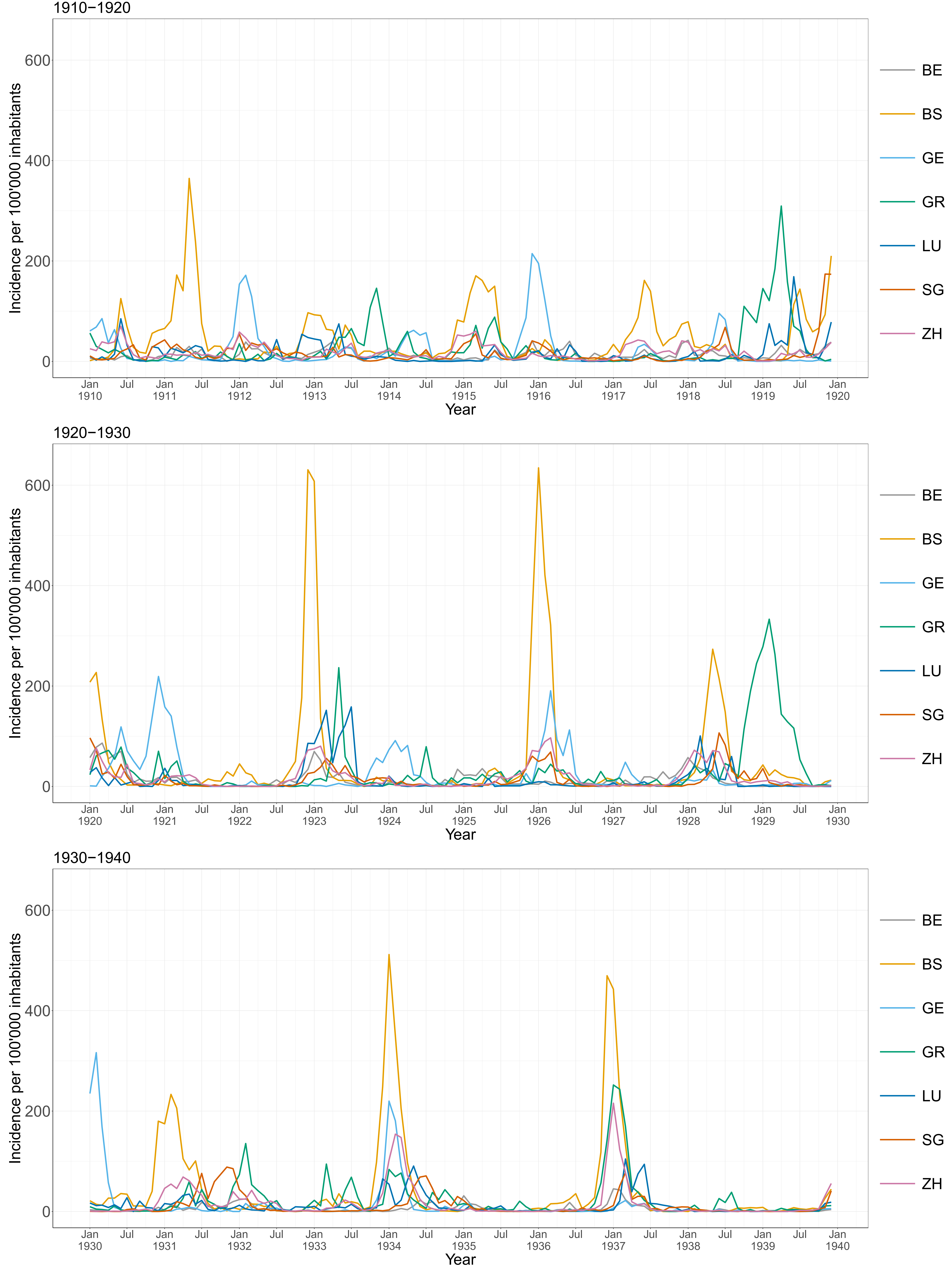


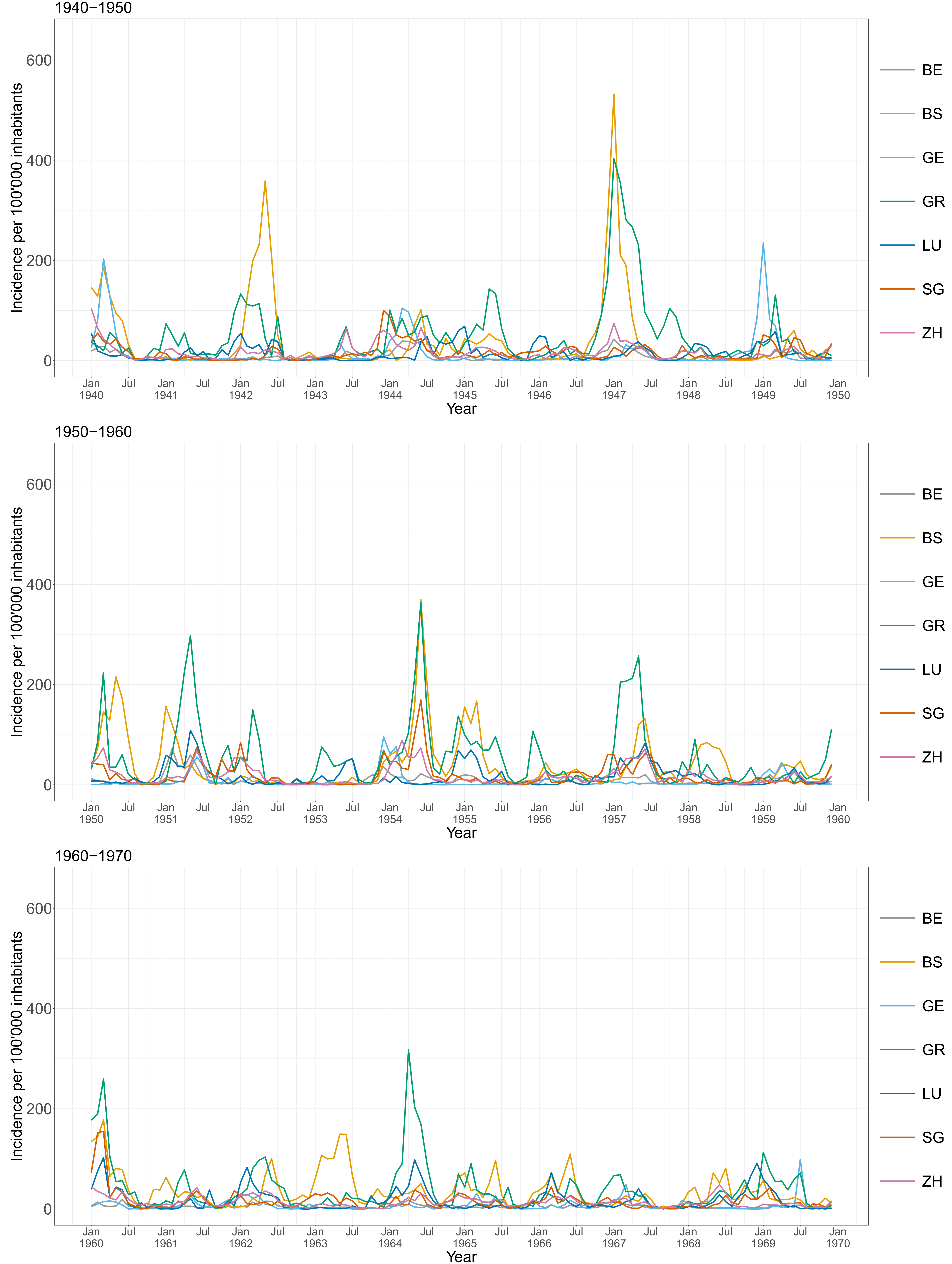


**Figure S3:** Wavelet power spectrum of measles incidence of the selected cantons of Zurich (ZH), Bern (BE), Lucerne (LU), St Gallen (SG), Grisons (GR), Basel-City (BS) and Geneva (GE) individually, as well as the total for the selected cantons combined. The y-axis follows a logarithmic progression.


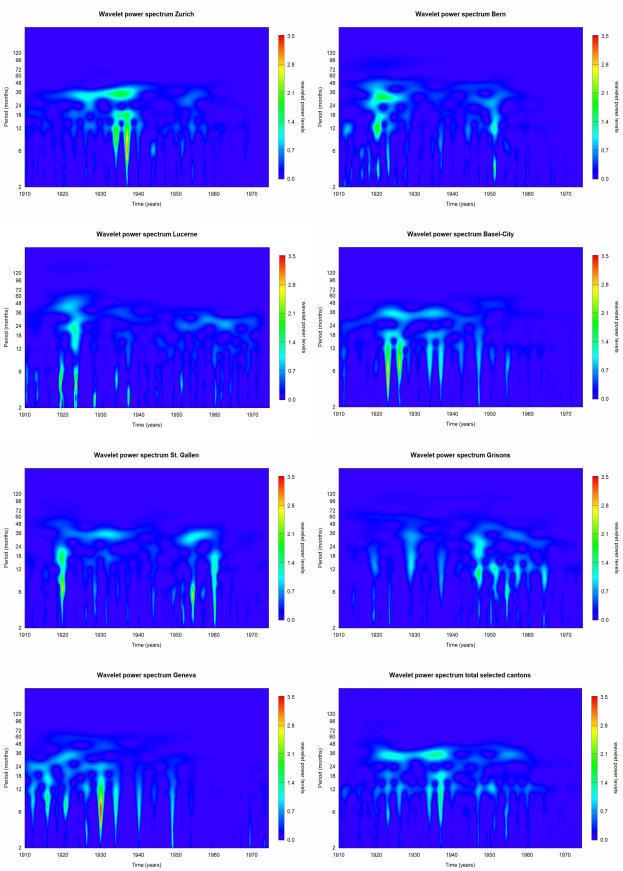


Figure S4: Average cross-wavelet power of measles incidence of all possible pairs of selected cantons (Zurich (ZH), Bern (BE), Lucerne (LU), St Gallen (SG), Grisons (GR), Basel-City (BS) and Geneva (GE)). The red lines indicate the periods of 12, 24 and 36 months. The y-axis follows a logarithmic progr**e**ssion.


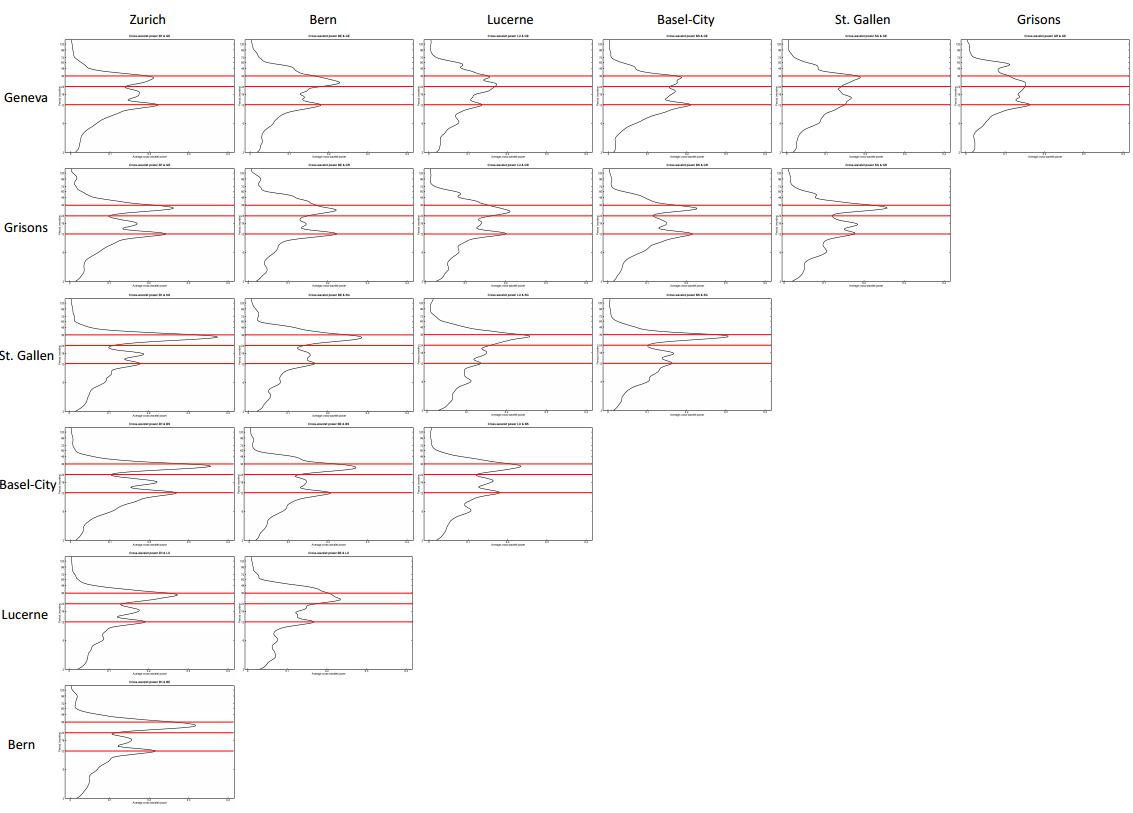


Figure S5: Cross-wavelet power spectrum of measles incidence of all possible pairs of the selected cantons (Zurich (ZH), Bern (BE), Lucerne (LU), St Gallen (SG), Grisons (GR), Basel-City (BS) and Geneva (GE)). The red lines indicate the periods of 12, 24 and 36 months. The y-axis follows a logarithmic progr**e**ssion


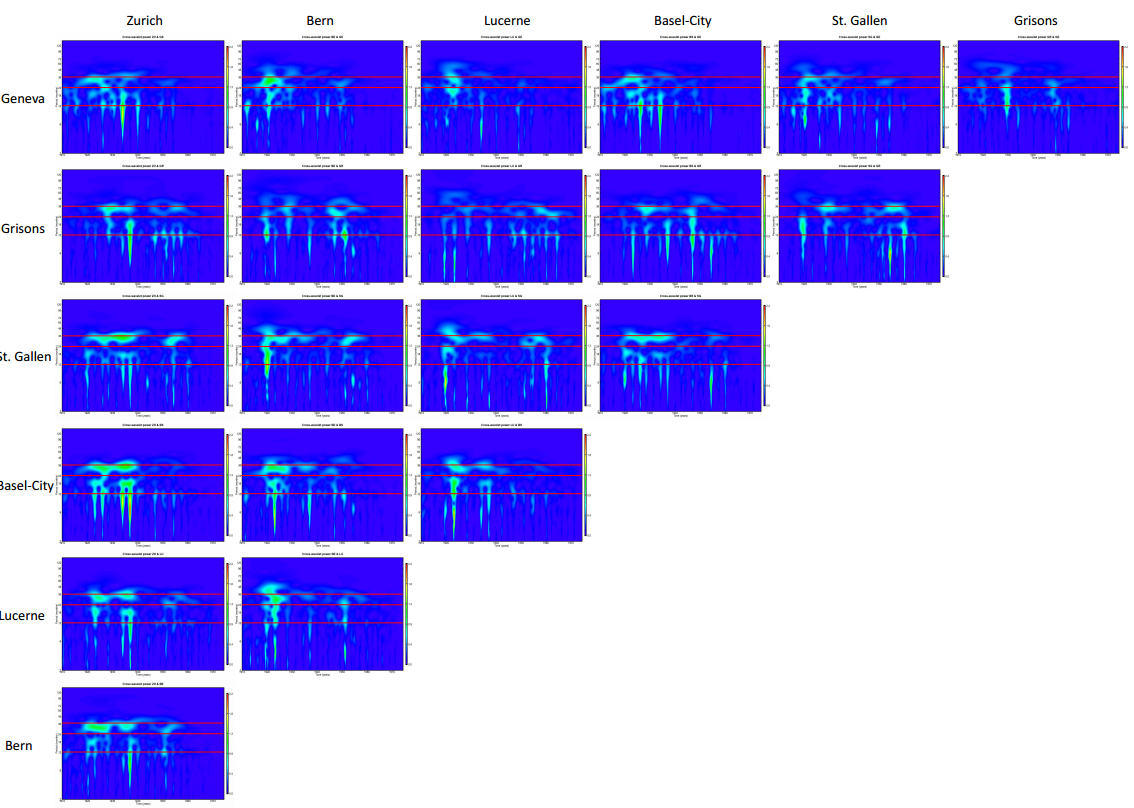

Supplement: Web_Material_kwaf167 [file web_material_kwaf167.zip › Supplementary Material.docx]
